# Microbiome analyses of 12 psyllid species of the family Psyllidae identified various bacteria including *Fukatsuia* and *Serratia symbiotica*, known as secondary symbionts of aphids

**DOI:** 10.1186/s12866-021-02429-2

**Published:** 2022-01-07

**Authors:** Atsushi Nakabachi, Hiromitsu Inoue, Yuu Hirose

**Affiliations:** 1 Electronics-Inspired Interdisciplinary Research Institute (EIIRIS), Toyohashi University of Technology, 1-1 Hibarigaoka, Tempaku, Toyohashi, Aichi 441-8580 Japan; 2 Department of Applied Chemistry and Life Sciences, Toyohashi University of Technology, 1-1 Hibarigaoka, Tempaku, Toyohashi, Aichi 441-8580 Japan; 3 Institute for Plant Protection, National Agriculture and Food Research Organization, Higashihiroshima, Hiroshima, 739-2494 Japan

**Keywords:** Psyllinae, Macrocorsinae, *Liberibacter*, *Wolbachia*, *Rickettsia*, *Diplorickettsia*, *Fukatsuia*, *Serratia symbiotica*, *Sodalis*, *Arsenophonus*

## Abstract

**Background:**

Psyllids (Hemiptera: Psylloidea) comprise a group of plant sap-sucking insects that includes important agricultural pests. They have close associations not only with plant pathogens, but also with various microbes, including obligate mutualists and facultative symbionts. Recent studies are revealing that interactions among such bacterial populations are important for psyllid biology and host plant pathology. In the present study, to obtain further insight into the ecological and evolutionary behaviors of bacteria in Psylloidea, we analyzed the microbiomes of 12 psyllid species belonging to the family Psyllidae (11 from Psyllinae and one from Macrocorsinae), using high-throughput amplicon sequencing of the 16S rRNA gene.

**Results:**

The analysis showed that all 12 psyllids have the primary symbiont, *Candidatus* Carsonella ruddii (Gammaproteobacteria: Oceanospirillales), and at least one secondary symbiont. The majority of the secondary symbionts were gammaproteobacteria, especially those of the family Enterobacteriaceae (order: Enterobacteriales). Among them, symbionts belonging to “endosymbionts3”, which is a genus-level monophyletic group assigned by the SILVA rRNA database*,* were the most prevalent and were found in 9 of 11 Psyllinae species. *Ca*. Fukatsuia symbiotica and *Serratia symbiotica*, which were recognized only as secondary symbionts of aphids, were also identified. In addition to other Enterobacteriaceae bacteria, including *Arsenophonus*, *Sodalis*, and “endosymbionts2”, which is another genus-level clade, *Pseudomonas* (Pseudomonadales: Pseudomonadaceae) and *Diplorickettsia* (Diplorickettsiales: Diplorickettsiaceae) were identified. Regarding Alphaproteobacteria, the potential plant pathogen *Ca*. Liberibacter europaeus (Rhizobiales: Rhizobiaceae) was detected for the first time in *Anomoneura mori* (Psyllinae), a mulberry pest. *Wolbachia* (Rickettsiales: Anaplasmataceae) and *Rickettsia* (Rickettsiales: Rickettsiaceae), plausible host reproduction manipulators that are potential tools to control pest insects, were also detected.

**Conclusions:**

The present study identified various bacterial symbionts including previously unexpected lineages in psyllids, suggesting considerable interspecific transfer of arthropod symbionts. The findings provide deeper insights into the evolution of interactions among insects, bacteria, and plants, which may be exploited to facilitate the control of pest psyllids in the future.

**Supplementary Information:**

The online version contains supplementary material available at 10.1186/s12866-021-02429-2.

## Background

Jumping plant lice or psyllids (Hemiptera: Sternorrhyncha: Psylloidea) are plant sap-sucking insects comprising about 4000 described species in the world [1, 2]. As with other sternorrhynchan insects [3–5], psyllids feed exclusively on phloem sap throughout their life [1, 2, 6]. Due to this feeding habit, some species transmit plant pathogens, including *Candidatus* Liberibacter spp. (Alphaproteobacteria: Rhizobiales) and *Ca*. Phytoplasma spp. (Bacilli: Acholeplasmatales), which makes them notorious agricultural or horticultural pests [7–9]. Also, as the phloem sap diet is deficient in nutrients including essential amino acids and some vitamins [10–12], psyllids depend on vertically transmitted bacterial mutualists to compensate for the nutritional deficiency. They typically harbor two distinct symbionts [13–31] within a specialized abdominal organ called the bacteriome [32]. The ‘primary symbiont’, assumed to be present in all psyllid species [13–16, 18–31, 33–35], is *Ca*. Carsonella ruddii (Gammaproteobacteria: Oceanospirillales) [33], which provides the host with essential amino acids [21, 22, 31, 35]. Molecular phylogenetic analyses demonstrated cospeciation between psyllids and *Carsonella*, resulting from a single acquisition of a *Carsonella* ancestor by a psyllid common ancestor and its stable vertical transmission since then [16, 19, 25, 33, 34]. Another bacterial lineage harbored in the bacteriome is categorized as a ‘secondary symbiont’. The secondary symbionts are phylogenetically diverse among psyllid species and genera, suggesting repeated infections and replacements during the evolution of Psylloidea [14, 16, 17, 19, 21, 24, 25, 27, 28]. Although secondary symbionts in various insect hosts range from parasites to mutualists [36–44], those in the psyllid bacteriome appear consistently to have obligate mutualistic features like the primary symbionts [21, 22, 24–26, 28, 31]. Because such features are characteristic of nutritional symbionts [36, 45–53], secondary symbionts in the psyllid bacteriome are generally believed to have a nutritional basis [19, 26, 28, 29]*,* as confirmed in *Ctenarytaina eucalypti* (Aphalaridae: Spondyliaspidinae) and *Heteropsylla cubana* (Psyllidae: Ciriacreminae) [21]. A unique exception is *Ca*. Profftella armatura (Gammaproteobacteria: Burkholderiales) found in psyllids of the genus *Diaphorina* (Psyllidae: Diaphorininae) [22, 30, 31, 54], whose main role appears to be protection of the holobiont (host + symbionts) from natural enemies [22, 31, 55–57]. In addition to these bacteriome-associated obligate mutualists, psyllids may harbor various secondary symbionts of a facultative nature, including *Wolbachia* (Alphaproteobacteria: Rickettsiales), *Rickettsia* (Alphaproteobacteria: Rickettsiales), *Rickettsiella* (Gammaproteobacteria: Diplorickettsiales), and *Diplorickettsia* (Gammaproteobacteria: Diplorickettsiales), which can cause systemic infection in the host insects [19–21, 23, 24, 26, 28, 29]. Moreover, recent studies are revealing that interactions among psyllid bacterial populations, including those associated with the bacteriome, facultative symbionts, and plant pathogens, are important for psyllid biology and host plant pathology [58–62]. Thus, elucidating microbiomes of various psyllid lineages, which reflect the ecological and evolutionary behaviors of bacterial populations in Psylloidea, would guide strategies to better control pest species.

According to the definition recently revised by Burckhardt et al. [2], psyllids are classified into seven extant families: Aphalaridae, Calophyidae, Carsidaridae, Liviidae, Mastigimatidae, Psyllidae, and Triozidae. Among them, Psyllidae is the most species-rich family (1381 species), whose largest constituent is the subfamily Psyllinae (795 species) [63]. Whereas several high-throughput amplicon-sequencing analyses have been performed on psyllid microbiomes, the target psyllids were biased toward the two most devastating pests *Diaphorina citri* (Psyllidae: Diaphorininae) and *Bactericera cockerelli* (Triozidae) as well as Aphalaridae species [23, 24, 27–30, 64–66]. Although recently published study analyzed psyllids from five families, the analysis was based on clustering sequences with a similarity threshold of 97%, resulting in a lower resolution [67]. In the present study, Illumina sequencing of 16S rRNA genes followed by resolving sequence variants down to the level of single-nucleotide differences was performed to assess the microbiomes of 12 Psyllidae species collected in Japan, focusing especially on Psyllinae (Table 1). Whereas these psyllids include agricultural pests (*Anomoneura mori* for the mulberry; *Cacopsylla biwa* for the loquat; *Cacopsylla burckhardti* and *Cacopsylla jukyungi* for the pear; and *Cacopsylla coccinea* for the akebi), none are known to vector plant pathogens. However, some other *Cacopsylla* spp. transmit phytoplasmas [7], and recent analyses have detected potentially pathogenic microbes from unexpected psyllid species [29, 30, 67]. Thus, the present study was performed to elucidate the ecological and evolutionary behaviors of various bacteria in psyllids, aiming to facilitate better pest management in the future.

**Table 1:** Psyllid species used for the present study

Species	Subfamily	Sampling site	Collection date	Host plant
*Anomoneura mori* Schwarz	Psyllinae	Banshoin, Izuhara, Tsushima City, Nagasaki Pref., Tsushima Isls, Japan	28/05/2013	*Morus* sp. (Moraceae)
*Cacopsylla biwa* Inoue	Psyllinae	Ikuna, Katsuura, Tokushima Pref., Shikoku, Japan	27/11/2013	*Eriobotrya japonica* (Rosaceae)
*Cacopsylla burckhardti* Luo et al.	Psyllinae	Shimoichida, Takamori, Nagano Pref., Honshu, Japan	23/05/2012	*Pyrus calleryana* (Rosaceae)
*Cacopsylla coccinea* (Kuwayama)	Psyllinae	Hayasaki, Kuchinotsu-chô, Minamishimabara City, Nagasaki Pref., Kyushu, Japan	08/04/2015	*Akebia quinata* (Lardizabalaceae)
*Cacopsylla fatsiae* (Jensen)	Psyllinae	Mt. Kadoyama, Fukuregi, Amakusa City, Kumamoto Pref., Amakusa-shimoshima Is., Kyushu, Japan	26/05/2015	*Fatsia japonica* (Araliaceae)
*Cacopsylla jukyungi* (Kwon)	Psyllinae	Taniguchi, Minamihata, Imari City, Saga Pref., Kyushu, Japan	10/08/2011	*Pyrus pyrifolia* var. *culta* (Rosaceae)
*Cacopsylla kiushuensis* (Kuwayama)	Psyllinae	Nodahama, Kazusa-chô, Minamishimabara City, Nagasaki Pref., Kyushu, Japan	30/04/2015	*Elaeagnus pungens* (Elaeagnaceae)
*Cacopsylla peninsularis* (Kwon)	Psyllinae	Notôge, Saigawa-hobashira, Miyako-machi, Fukuoka Pref., Kyushu, Japan	20/05/2015	*Sorbus japonica* (Rosaceae)
*Cacopsylla satsumensis* (Kuwayama)	Psyllinae	Kôtsufukae, Reihoku-machi, Kumamoto Pref., Amakusa-shimoshima Is., Kyushu, Japan	09/04/2015	*Rhaphiolepis indica* var. *umbellata* (Rosaceae)
*Cyamophila hexastigma* (Horvath)	Psyllinae	Jozankei Dam, Sapporo City, Hokkaido, Japan	12/06/2013	*Maackia amurensis* (Fabaceae)
*Psylla morimotoi* Miyatake	Psyllinae	Ooi, Shinano, Nagano Pref., Honshu, Japan	16/06/2008	*Prunus grayana* (Rosacae)
*Epiacizzia kuwayamai* (Crawford)	Macrocorsinae	Koba, Obama-chô, Unzen City, Nagasaki Pref., Kyushu, Japan	30/04/2015	*Neolitsea sericea* (Lauraceae)

## Results and discussion

### All 12 Psyllidae species have *Carsonella* and at least one other symbiont

**Fig. 1:**
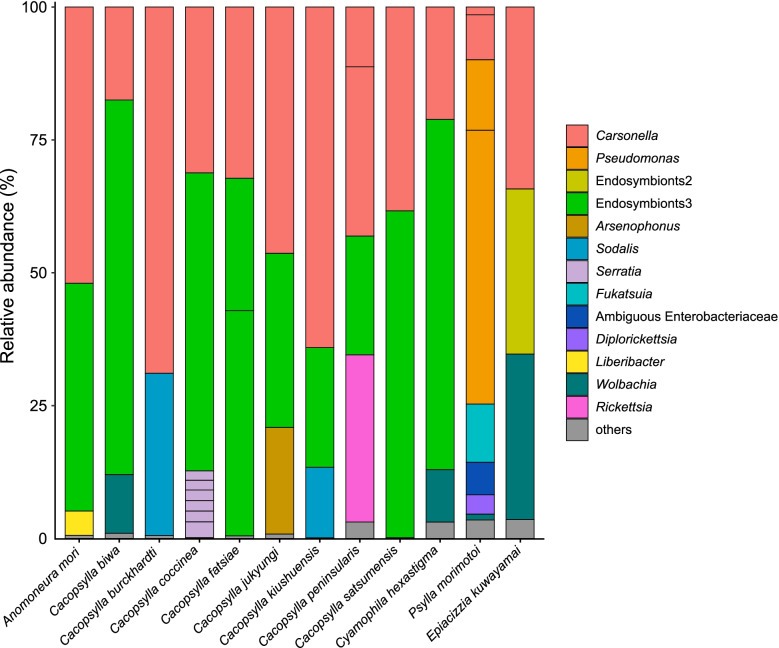
Composition of bacterial populations in psyllids of the family Psyllidae. Relative abundances of Illumina reads belonging to assigned bacterial taxa are shown

**Fig. 2:**
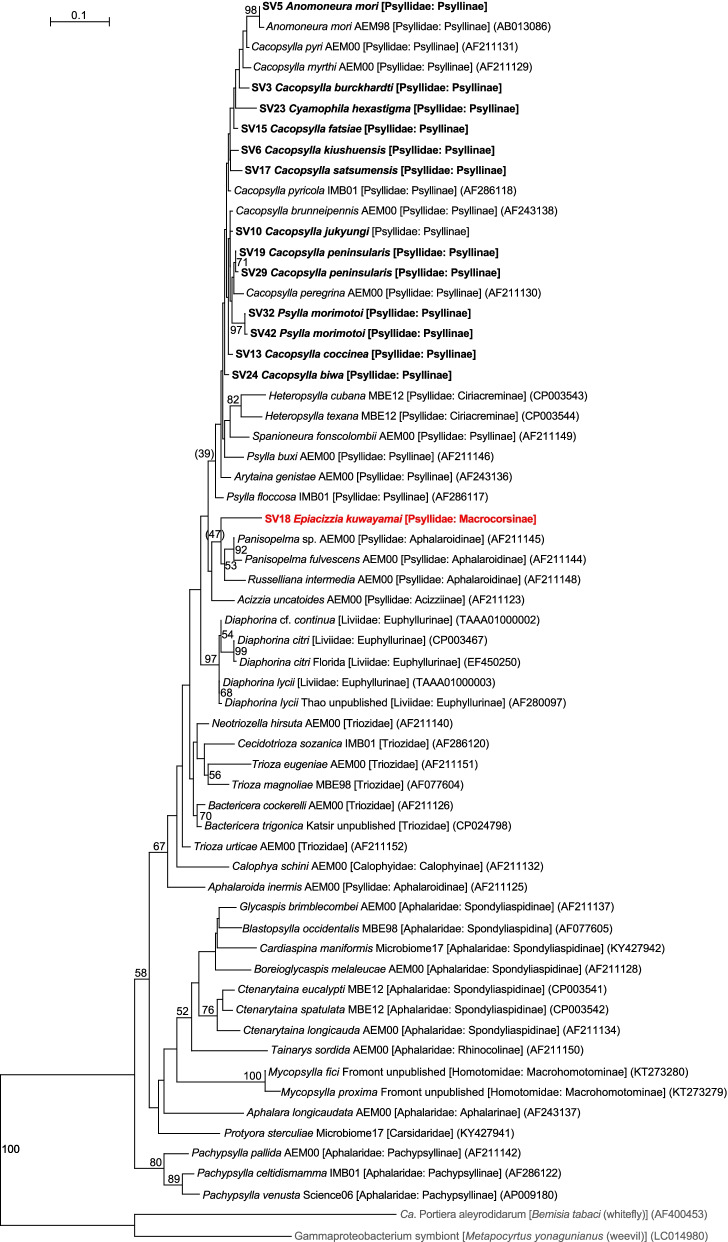
Maximum likelihood phylogram of *Carsonella*. A total of 427 aligned nucleotide sites of 16S rRNA genes were subjected to the analysis. On each branch, bootstrap support values of > 50% are shown. Designations other than those for outgroups refer to psyllid hosts. Families and subfamilies (if applicable) of the host psyllids are shown in brackets. Sequences from this study are shown in bold. DDBJ/EMBL/GenBank accession numbers for sequences are provided in parentheses. The sequence from *E. kuwayamai* is highlighted in red. The bar represents nucleotide substitutions per position. The outgroups were *Ca*. Portiera aleyrodidarum; the primary symbiont of the whitefly *Bemisia tabaci* (Hemiptera: Sternorrhyncha: Aleyrodoidea), and a gammaproteobacterium symbiont of the weevil *Metapocyrtus yonagunianus* (Coleoptera: Curculionidae)

MiSeq sequencing of the amplicon libraries yielded 46,568–73,470 pairs of forward and reverse reads for the 12 psyllid species (Supplementary Table 1). Denoising and joining of the paired-end reads along with removal of low-quality or chimeric reads resulted in 37,901–63,866 non-chimeric high-quality reads (Supplementary Table 1). Dereplication of these reads resulted in 207 independent sequence variants (SVs), among which only 43 SVs accounted for > 1% of the total reads (Supplementary Table 2). We focused on these 43 SVs, because the targets of the present study were relatively abundant symbionts with close association with the host psyllids, and filtering with the threshold of 1% was shown to be among the most effective and accurate methods to remove potential contaminants derived from environments and experimental reagents [68]. SVs with a relative abundance of less than 1% are collectively categorized as ‘others’ in Fig. 1, which correspond to 0.16 – 3.56% reads in total in each psyllid species (Supplementary Table 2). Notably simple bacterial communities like these have been reported for sternorrhynchan insects with bacteriomes, including aphids, whiteflies, and other psyllid species [24, 28, 30, 37, 64–66, 69]. All the SVs with a relative abundance of greater than 1% were highly similar to the sequences that were reported to be of insect symbionts (see below). Taxonomic classification by QIIME2 (Supplementary Table 2) followed by independent BLAST searches and phylogenetic analyses showed that all the 12 psyllid species possess distinct lineages of *Carsonella* (Fig. 1). Because *Carsonella* has been repeatedly shown to be cospecified with host psyllids [16, 19, 25, 33, 34], the phylogenetic relationship of *Carsonella* is assumed to be useful to infer that of the host psyllids. In the maximum likelihood (ML) tree, the *Carsonella* sequences from Psyllinae species formed a clade with those of psyllids belonging to the subfamily Ciriacreminae, and the sequence from *Epiacizzia kuwayamai* formed an independent clade with those of Aphalaroidinae species (Fig. 2). The exclusion of *E. kuwayamai* from Psyllinae is consistent with the current classification of this species to the subfamily Macrocorsinae*.* However, these clades were only poorly supported by bootstrap values (47% for the *E. kuwayamai*-Aphalaroidinae clade and 39% for the Psyllinae-Ciriacreminae clade), requiring further studies to clarify the phylogenetic position of *E. kuwayamai.* Besides, the SVs from eight *Cacopsylla* species did not form a clade (Fig. 2), implying their polyphyly as presumed by Burckhardt et al. [2]. However, this branching pattern also lacked robust statistical support (< 50%). Two types each of *Carsonella* sequences were detected in *Cacopsylla peninsularis* and *Psylla morimotoi* (Fig. 1, Supplementary Table 2). In *C. peninsularis*, SV19 and SV29 were 99.8% identical (Supplementary Table 2) and formed a clade supported by a bootstrap value of 71% (Fig. 2). SV32 and SV42 from *P. morimotoi* were also 99.8% identical (Supplementary Table 2), forming a clade supported by a bootstrap value of 97% (Fig. 2). These may reflect sequence variations in each lineage of *Carsonella*. Although we cannot exclude the possibility that they are artifacts due to polymerase chain reaction (PCR)/sequencing errors, the latter seems less likely because the dada2 plugin corrects sequencing errors during the denoising process [70, 71]. Some previous studies that analyzed psyllid microbiomes using ‘universal primers’ detected only a trace amount of *Carsonella* reads [27–29, 64, 66, 67]; however, the present study, which used primers appropriately modified to improve sensitivity to highly AT-biased symbiont genes [21, 22, 31, 35], detected a large percentage of *Carsonella* reads (Fig. 1, Supplementary Table 2), which reflects actual populations more precisely [30].

Besides *Carsonella*, all 12 psyllids analyzed in the present study possessed at least one other symbiont (Fig. 1).

### Various Enterobacteriaceae bacteria reside in Psyllidae

Of the 43 SVs obtained in the present study, 39 corresponded to gammaproteobacteria, among which 22 belonged to the family Enterobacteriaceae (order Enterobacteriales) (Supplementary Table 2). Enterobacteriaceae is a group of bacteria that encompasses an especially large fraction of intimate insect symbionts, including those associated with the bacteriome [36, 46]. Enterobacteriaceae bacteria identified in the present study include *Arsenophonus*, *Fukatsuia*, *Serratia*, *Sodalis*, endosymbionts2, and endosymbionts3. Among them, the most prevalent was endosymbionts3, which is a genus-level monophyletic group of endosymbionts assigned by the SILVA rRNA database project [72].

### Prevalent endosymbionts3

**Fig. 3:**
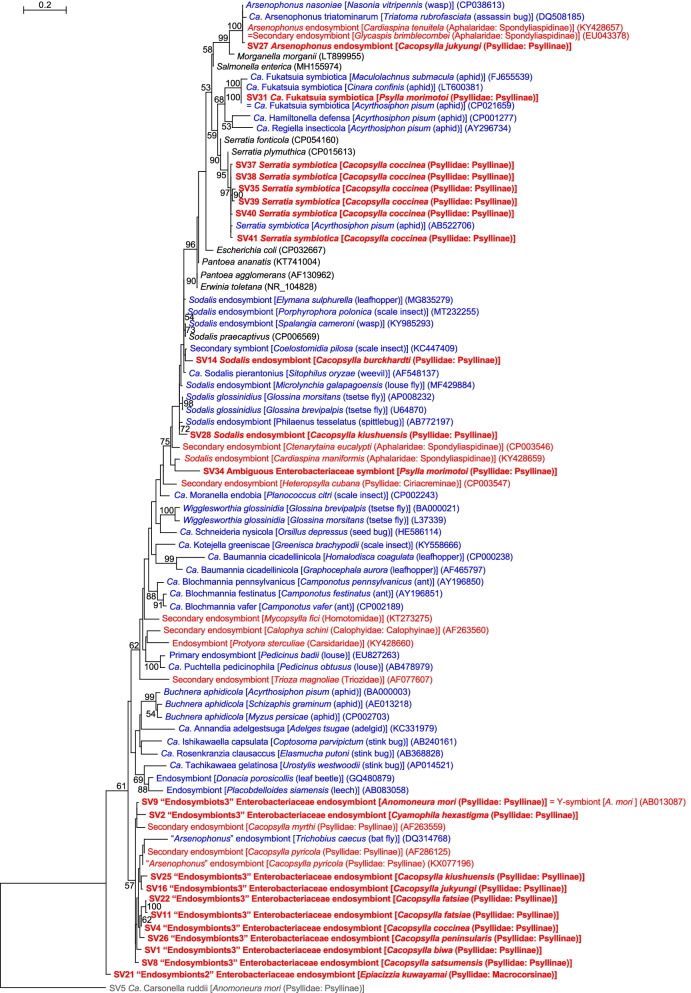
Maximum likelihood phylogram of bacteria belonging to Enterobacteriaceae. A total of 428 unambiguously aligned nucleotide sites of 16S rRNA genes were subjected to the analysis. On each branch, bootstrap support values of > 50% are shown. The scale bar indicates substitutions per site. For symbiotic bacteria, host organisms are shown in brackets. Symbionts of animals other than psyllids are shown in blue. Symbionts of psyllids are shown in red. Sequences from this study are shown in bold. DDBJ/EMBL/GenBank accession numbers are provided in parentheses. *Carsonella* was used as an outgroup

Ten SVs corresponding to distinct lineages of endosymbionts3 were detected in 9 of 11 Psyllinae species (Fig. 1, Supplementary Table 2). Namely, one SV each for endosymbionts3 was observed in *Anomoneura mori* (SV9: 42.9% of the total denoised reads in *A. mori*), *Cacopsylla biwa* (SV1: 70.5% of the *C. biwa* reads), *Cacopsylla coccinea* (SV4: 56.1% of the *C. coccinea* reads), *Cacopsylla jukyungi* (SV16: 32.8% of the *C. jukyungi* reads), *Cacopsylla kiushuensis* (SV25: 22.5% of the *C. kiushuensis* reads), *Cacopsylla peninsularis* (SV26: 22.4% of the *C. peninsularis* reads), *Cacopsylla satsumensis* (SV8: 61.5% of the *C. satsumensis* reads), and *Cyamophila hexastigma* (SV2: 65.9% of the *Cy. hexastigma* reads). Two SVs corresponding to endosymbionts3, which may reflect sequence variations, were detected in *Cacopsylla fatsiae* (SV11 and SV22: 42.4 and 24.9%, respectively, of the *C. fatsia* reads). These SVs were 93.4% (SV2 vs SV11) − 99.8% (SV11 vs SV22) identical to one another. SV9 was 100% identical to the ‘Y-symbiont’ sequence of *A. mori* (AB013087), which was previously detected via cloning methods [15]. The other nine SVs were 96.2% − 97.7% identical to the sequences of the “*Arsenophonus*” symbionts of *Cacopsylla pyricola* (Psyllidae: Psyllinae) (KX077196) and the bat fly *Trichobius caecus* (Diptera: Streblidae) (DQ314768) [73]. These sequences formed a moderately supported clade (bootstrap: 57%) in the ML tree (Fig. 3). Although these references were named “*Arsenophonus*”, they were only 84.3% − 87.7% identical to the sequence of the type species *Arsenophonus nasoniae* (CP038613) [74], and were excluded from the robustly supported clade (bootstrap: 100%) formed by *Arsenophonus nasoniae*, *Ca*. Arsenophonus triatominarum, and SV27, assigned as *Arsenophonus* by QIIME2 in the present study (see below) (Fig. 3). Moreover, this *Arsenophonus nasoniae* clade formed a strongly supported clade (bootstrap: 96%) with other well-known insect symbionts, including *Fukatsuia*, *Hamiltonella*, *Regiella*, and *Serratia*, excluding the clade of endosymbionts3 (Fig. 3). These findings may suggest reconsideration of the naming of “*Arsenophonus*” symbionts that clustered with the endosymbionts3 bacteria. Although little is known about the functions of endosymbionts3-type symbionts [72], the prevalence among analyzed psyllids, high abundance of corresponding reads within each psyllid, and relatively low G + C% (< 50%) of the reads (Fig. 1, Supplementary Table 2) suggest that endosymbionts3 are ancient bacteriome-associated secondary symbionts in these psyllid lineages, which potentially complement the partially deficient functions of *Carsonella*.

### *Arsenophonus* symbiont

In addition to *Carsonella* and an endosymbionts3 bacterium, *Arsenophonus* was observed in *C. jukyungi* (Fig. 1). Specifically, QIIME2 assigned SV27, which was derived from 20.0% of denoised *C. jukyungi* reads (Supplementary Table 2), to *Arsenophonus*. SV27 was 99.5% identical to the sequence of *Arsenophonus* symbionts of a wide variety of insects, including other psyllid species, *Cardiaspina tenuitela* (Aphalaridae: Spondyliaspidinae) (KY428657) and *Glycaspis brimblecombei* (Aphalaridae: Spondyliaspidinae) (EU043378) [25, 28]. It was 99.3% identical to *Arsenophonus nasoniae* (CP038613), the type species of *Arsenophonus* found in the parasitoid wasp *Nasonia vitripennis* (Hymenoptera: Pteromalidae), and 98.4% identical to *Ca*. Arsenophonus triatominarum (DQ508185) found in the assassin bug *Triatoma rubrofasciata* (Hemiptera: Reduviidae). As mentioned above, SV27 formed a robustly supported clade (bootstrap: 100%) with these *Arsenophonus* symbionts (Fig. 3). Whereas *Arsenophonus* shows a wide range of associations from parasitic to obligately mutualistic to the host insects [74, 75], its ecological role in psyllids is currently unknown.

### Detection of *Ca*. Fukatsuia symbiotica in psyllids

SV31, which was derived from 11.0% of denoised *P. morimotoi* reads, was 100% identical to *Ca*. Fukatsuia symbiotica (CP021659) of the pea aphid *Acyrthosiphon pisum* (Hemiptera: Sternorrhyncha: Aphidoidea: Aphididae) (Fig. 1, Supplementary Table 2). It was 99.3 and 98.6% identical to *Fukatsuia* from other aphid species, *Cinara confinis* (Aphididae) (LT600381) and *Maculolachnus submacula* (Aphididae) (FJ655539), respectively. SV31 formed a robustly supported clade (bootstrap: 100%) with these sequences in the ML tree (Fig. 3). To our knowledge, this is the first formal report of *Fukatsuia* detected in psyllids. *Fukatsuia* has only been recognized as a secondary symbiont of aphids, with a wide variety of reported roles, including pathogen, parasite, defensive symbiont, and obligate nutritional symbiont [76]. The recently revealed culturability of *Fukatsuia* [76] indicates its ability to survive outside aphids, which would facilitate horizontal transfer to other insects, including psyllids. It would be interesting to assess the prevalence and functional role of *Fukatsuia* in Psylloidea.

### Detection of *Serratia symbiotica* in psyllids

Six SVs found in *C. coccinea* corresponded to the sequence of *Serratia symbiotica*, known as a prevalent secondary symbiont of aphids. Namely, SV35, SV37, SV38, SV39, SV40, and SV41, which accounted for 3.0, 2.0, 2.0, 2.0, 1.8, and 1.8% of the denoised *C. coccinea* reads, respectively, were 98.8 – 99.8% identical to a single sequence of *S. symbiotica* (AB522706) (Fig. 1, Supplementary Table 2). This reference sequence was derived from various aphid lineages, including *Acyrthosiphon pisum*, *Aphis fabae*, *Aphis gossypii*, *Cinara pinikoraiensis*, *Cinara ponderosae*, and *Trama caudata* (all Aphididae). The SVs and *S. symbiotica* sequence from aphids formed a robustly supported clade (bootstrap: 97%) in the ML tree (Fig. 3). To our knowledge, this is the first formal report of *S. symbiotica* or its close relative detected in psyllids, although there was a previous mention with no concrete data [77]. These SVs were 98.4% (SV35 vs SV41) – 99.8% (SV38 vs SV40) identical to one another. The similarities both in nucleotide sequences and read frequencies imply that the SVs correspond to multiple copies of the 16S rRNA gene in a single *S. symbiotica* genome. This is consistent with the fact that genomes of several *S. symbiotica* strains encode more than a single copy of the 16S rRNA gene [42, 43], which contrasts the case of primary symbionts with an extremely streamlined genome encoding only a single copy. Similar to *Fukatsuia*, the ecological role of *S. symbiotica* is reported to be widely varied depending on aphid lineages [43, 44]; however, its role in psyllids is currently unknown. Further studies are required to assess this aspect. As Pons et al. showed that *S. symbiotica* can enter plants and cause new infection in aphids, host plants are likely media for intra-and interspecific horizontal transmission of this bacterium [77].

### *Sodalis* symbionts and its relative

*Sodalis* endosymbionts were detected in *C. burckhardti* and *C. kiushuensis* (Fig. 1, Supplementary Table 2). SV14, which was derived from 30.5% of the *C. burckhardti* reads, was 96.3% identical to the sequence of the type species *Sodalis glossinidius* (AP008232), a secondary symbiont of the tsetse fly *Glossina morsitans* (Diptera: Glossinidae). The sequence was 96.7% identical to that of *Ca*. Sodalis pierantonius (AF548137), the primary symbiont of the rice weevil *Sitophilus oryzae* (Coleoptera: Curculionidae). The sequence was 95.6 – 97.9% identical to those of *Sodalis* endosymbionts from various insects. SV28, which was derived from 13.3% of the *C*. *kiushuensis* reads, was 96.7% identical to the *Sodalis glossinidius* sequence. It was 96.0% identical to the sequence of *Ca*. Sodalis pierantonius, and 94.6 – 97.2% identical to those of the above-mentioned *Sodalis* endosymbionts from various insects. These sequences were clustered with that of a *Sodalis* endosymbiont from another psyllid *Cardiaspina maniformis* (Aphalaridae: Spondyliaspidinae) (KY428659) [28] and SV34 (see below), whose branching pattern was moderately supported (bootstrap:75%) (Fig. 3). *Sodalis* symbionts have been detected in a wide variety of insects and are known to have replaced more ancient predecessor symbionts in weevils (Coleoptera: Curculionoidea) [78] and spittlebugs (Hemiptera: Cercopoidea) [79]. In this context, the distribution of the *Sodalis* symbiont in *Cacopsylla* spp. may be of interest. Whereas endosymbionts3 appear dominant (presumably bacteriome-associated) secondary symbionts in *Cacopsylla* spp., *C. kiushuensis* additionally has a *Sodalis* symbiont, and *C. burckhardti* has only *Carsonella* and *Sodalis.* This might imply, though speculative, that replacement of endosymbionts3 by *Sodalis* is at initial stage in *C. kiushuensis*, and is completed in *C. burckhardti.*

Regarding SV34, which was derived from 6.1% of denoised *P. morimotoi* reads, QIIME2 failed to assign a genus-level taxonomy (Fig. 1, Supplementary Table 2). The BLAST best hit of SV34 was *Sodalis* endosymbiont of the psyllid *Cardiaspina maniformis* (Aphalaridae: Spondyliaspidinae) (KY428659) [28]. These sequences formed a cluster in the ML tree (Fig. 3). However, this branching pattern was only poorly supported (bootstrap: 31%), and their sequence identity was 93.0%, which was below the generally used arbitrary genus threshold of 94.5 – 95% [80, 81]. Thus, we refrained from assigning this symbiont to a particular genus.

### Putative endosymbionts2 symbiont

QIIME2 assigned SV21, which was derived from 31.1% of denoised *E. kuwayamai* reads, to endosymbionts2 (Fig. 1, Supplementary Table 2), another monophyletic group of endosymbionts assigned by SILVA [72]. The BLAST best hit of SV21 was a secondary endosymbiont of *Cacopsylla myrthi* (AF263559) [17], but the sequence identity was only 90.9%. SV21 branched basally to other Enterobacteriaceae bacteria in the ML tree (Fig. 3). It would be interesting to assess the prevalence of endosymbionts2 in the subfamily Macrocorsinae, in the context of the apparent prevalence of endosymbionts3 among Psyllinae species analyzed in the present study.

### *Psylla morimotoi* has *Pseudomonas* and *Diplorickettsia*

**Fig. 4:**
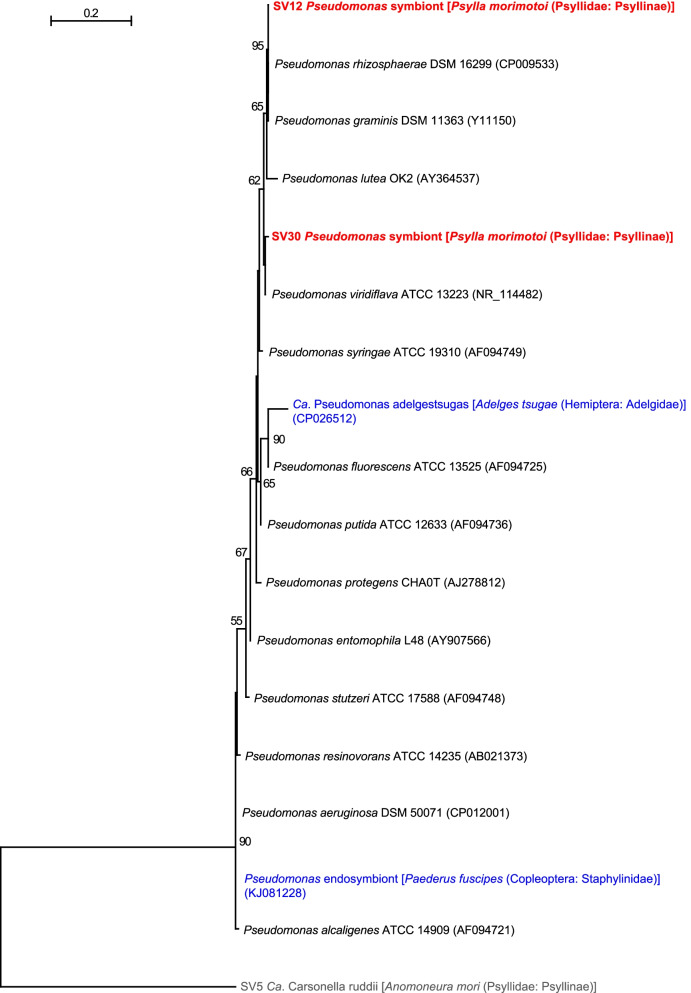
Maximum likelihood phylogram of *Pseudomonas*. A total of 427 unambiguously aligned nucleotide sites of 16S rRNA genes were subjected to the analysis. On each branch, bootstrap support values of > 50% are shown. The scale bar indicates substitutions per site. For symbiotic bacteria, host organisms are shown in brackets. Symbionts of animals other than psyllids are shown in blue. Sequences detected in the present study are shown in bold red. DDBJ/EMBL/GenBank accession numbers are provided in parentheses. *Carsonella* was used as an outgroup

Non-Enterobacteriales gammaproteobacteria found in the present study were *Carsonella* (Oceanospirillales: Halomonadaceae) mentioned above, *Pseudomonas* (Pseudomonadales: Pseudomonadaceae), and *Diplorickettsia* (Diplorickettsiales: Diplorickettsiaceae); of these, the latter two were detected from *P. morimotoi*. QIIME2 assigned SV12 and SV30, which were derived from 51.5 and 13.3% of denoised *P. morimotoi* reads, respectively (Fig. 1, Supplementary Table 2), to *Pseudomonas*. SV12 and SV30 shared 98.4% identity. SV12 was 100% identical to the sequences of various *Pseudomona*s strains, including type strains for *Pse. graminis* (Y11150) and *Pse. rhizosphaerae* (CP009533). SV30 was 99.5% identical to the sequence of the type strain of *Pse. viridiflava* (NR_114482). Although *Pseudomonas* species have been detected in various insects including psyllids, they are largely believed to be transient associates [28]. In contrast to Enterobacteriaceae bacteria, many of which have intimate and stable mutualistic relationships with insect hosts, known examples of *Pseudomonas* with such associations (vertically-transmitted endosymbionts present in the host hemocoel or cells) are limited in rove beetles (Coleoptera: Staphylinidae) [82] and the adelgid *Adelges tsugae* (Hemiptera: Sternorrhyncha: Phylloxeroidea: Adelgidae) [83]. Although SV12 and SV30 were not closely related to these symbionts (Fig. 4), the fact that the majority (64.8%) of reads in *P. morimotoi* corresponded to *Pseudomonas* (Fig. 1, Supplementary Table 2) implies that the *Pseudomonas* symbionts potentially play important roles in this psyllid.

**Fig. 5:**
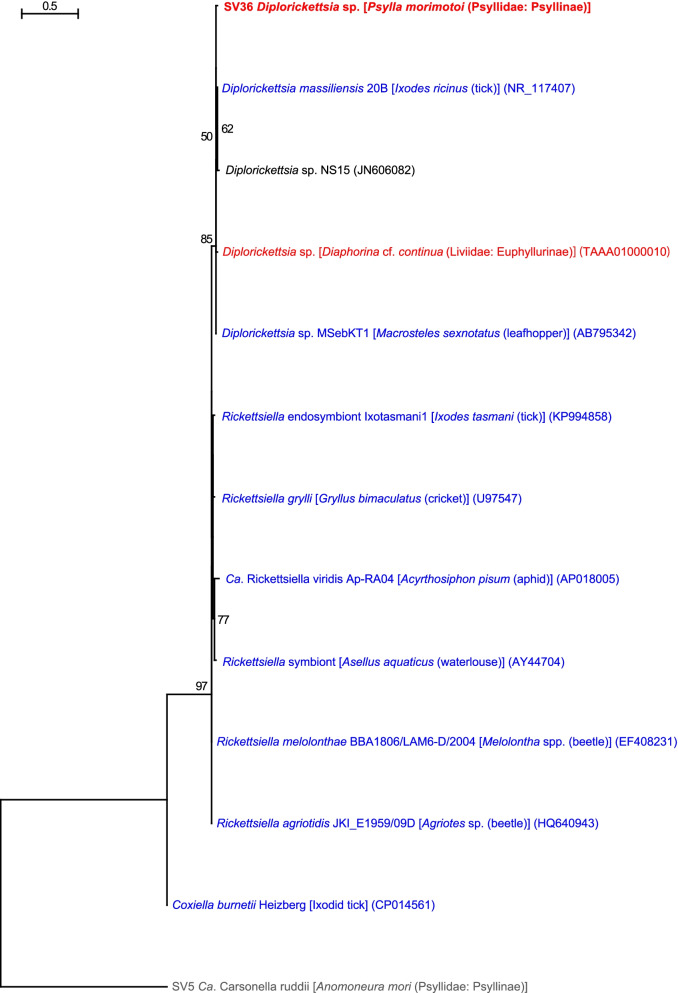
Phylogenetic position of *Diplorickettsia* lineages inferred by the maximum likelihood method. A total of 427 unambiguously aligned nucleotide sites of 16S rRNA genes were subjected to the analysis. On each branch, bootstrap support values of > 50% are shown. The scale bar indicates substitutions per site. For symbiotic bacteria, host organisms are shown in brackets. Symbionts of animals other than psyllids are shown in blue. Symbionts of psyllids are shown in red. The sequence from this study is shown in bold. DDBJ/EMBL/GenBank accession numbers are provided in parentheses. *Carsonella* was used as an outgroup

QIIME2 assigned SV36, which was derived from 3.7% of denoised *P. morimotoi* reads, to *Diplorickettsia* (Fig. 1, Supplementary Table 2). SV36 was 99.3% identical to the sequence of *Diplorickettsia massiliensis* 20B (NR_117407) detected in the European sheep tick *Ixodes ricinus* (Arachnida: Acari: Ixodidae), 98.8% identical to the sequence of *Diplorickettsia* sp. (TAAA01000010) recently found in another psyllid species *Diaphorina* cf. *continua* (Psyllidae: Diaphorininae) [30], 98.4% identical to that of *Diplorickettsia* sp. MSebKT1 (AB795342) detected in a leafhopper *Macrosteles sexnotatus* (Hemiptera: Auchenorrhyncha: Cicadellidae), and 98.1% identical to *Diplorickettsia* sp. NS15 (JN606082) found in human nasal samples. Molecular phylogenetic analysis showed that SV36 forms a well-supported clade (bootstrap: 85%) with these *Diplorickettsia* spp. (Fig. 5). *Diplorickettsia massiliensis* was observed in *Ixodes ricinus* and serum samples of human patients with suspected tick-borne disease, suggesting that this bacterium is a human pathogen [84, 85]. Subsequently, *Diplorickettsia* lineages were unexpectedly found in two plant sap-sucking hemipteran insects, *M. sexnotatus* collected in Japan [86]*,* and *D.* cf. *continua* collected in Corsica [30]. The present study adds another example of *Diplorickettsia*. These findings imply that *Diplorickettsia* is actually prevalent in various sap-sucking insects. Although their host plants are not shared among *M. sexnotatus* (Poaceae and Fabaceae), *D.* cf. *continua* (Thymelaeaceae), and *P. morimotoi* (Rosaceae), it would be worth assessing the possibility that the plants are also infected with *Diplorickettsia*. *Diplorickettsia* is closely related to the genus *Rickettsiella* (Diplorickettsiales: Diplorickettsiaceae) (Fig. 5) comprising intracellular bacteria associated with various arthropods, including insects, arachnids, and isopods [19, 28]. Whereas many *Rickettsiella* spp. are simply pathogenic to arthropods, *Ca*. Rickettsiella viridis [87] modifies the body color of aphids, potentially affecting the attractiveness of insects to natural enemies [88]. As little is known about the functions of *Diplorickettsia* in host arthropods, it would be interesting to assess the physiological and ecological effects of *Diplorickettsia* on psyllids.

### First detection of *Liberibacter* in *Anomoneura mori*

**Fig. 6:**
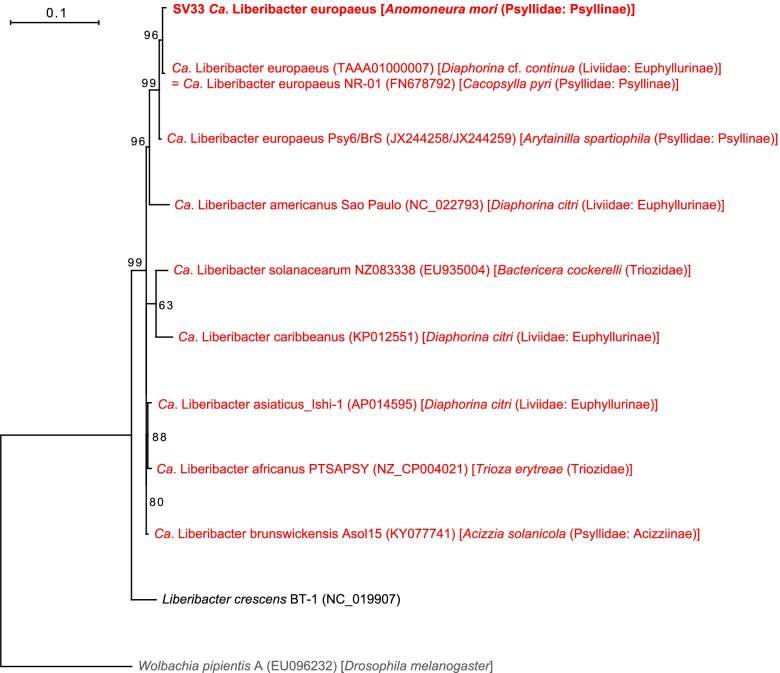
Maximum likelihood phylogram of *Liberibacter* spp. A total of 402 unambiguously aligned nucleotide sites of 16S rRNA genes were subjected to the analysis. On each branch, bootstrap support values of > 50% are shown. Symbionts of psyllids are shown in red. The sequence from this study is shown in bold. The host psyllids are shown in brackets. DDBJ/EMBL/GenBank accession numbers for sequences are provided in parentheses. The scale bar represents nucleotide substitutions per position. *Wolbachia* was used as an outgroup

The analysis detected *Ca*. Liberibacter europaeus (Alphaproteobacteria: Rhizobiales) for the first time in *A. mori*, a sericultural pest that feeds on the mulberry plants *Morus* spp. (Moraceae) (Fig. 1, Supplementary Table 2). Namely, SV33, which was derived from 4.6% of denoised *A. mori* reads, was 99.8% identical to the sequence of *Ca*. Liberibacter europaeus previously detected in *Cacopsylla pyri* (Psyllidae: Psyllinae) (FN678792) and *Diaphorina* cf. *continua* (Psyllidae: Diaphorininae) (TAAA01000007) [30]. Molecular phylogenetic analysis showed that these sequences form a robustly supported clade (bootstrap: 96%) within *Ca*. Liberibacter spp. (Fig. 6).

The genus *Liberibacter* currently includes nine species: *Ca*. L. asiaticus, *Ca*. L. americanus, and *Ca*. L. africanus, which cause greening disease in citrus (Rutaceae) [8, 89]; *Ca*. L. capsica, a potential pathogen of solanaceous plants [67]; *Ca*. L. caribbeanus found in citrus, but with uncertain pathogenicity [90]; *Ca*. L. solanacearum, which causes diseases in solanaceous and apiaceous plants [91–94]; *Ca*. L. brunswickensis, a probable endophyte of solanaceous plants [95]; *L. crescens*, which is non-pathogenic and the only culturable species in the genus [96]; and *Ca*. L. europaeus (*C*Leu) [97–99]. *C*Leu was detected from various psyllids in various locations: *Cacopsylla* spp. (Psyllidae: Psyllinae) in Italy and Hungary [97, 98], *Arytainilla spartiophila* (Psyllidae: Psyllinae) in New Zealand and the U.K. [99, 100], and *D*. cf. *continua* (Psyllidae: Diaphorininae) in Corsica island [30]. *C*Leu was also detected from rosaceous plants and the Scotch broom *Cytisus scoparius* (Fabaceae), which are host plants of *Cacopsylla* spp. and *Ar. spartiophila*, respectively [97–100]. Whereas the presence of *C*Leu is associated with pathological symptoms in the Scotch broom [99], no symptoms are known in rosaceous plants and *Thymelaea tartonraira* (Thymelaeaceae), the probable host plant of *D*. cf. *continua* [30]. The present study adds another example of *C*Leu from another psyllid species, *A. mori* (Psyllidae: Psyllinae) in Japan. Because *A. mori* is a pest species feeding on mulberry plants, it would be interesting to assess if the host plants, which are distantly related to previously known infected plants, are also infected with *C*Leu and whether infection causes symptoms of disease.

It appears that *Ca*. Liberibacter lineages have evolved in close association with Psylloidea, and all known vectors for all *Ca*. Liberibacter spp. are psyllids [8, 30, 67, 89–91, 94, 95, 97–99]. In this context, the finding that the fecundity and population growth rates of *D. citri* harboring *Ca*. L. asiaticus are increased as compared with uninfected insects [101] is particularly interesting. This observed benefit may be an ecological driver for the close association between *Ca*. Liberibacter spp. and psyllids. Future studies should focus on assessing the general applicability of this hypothesis to other *Ca*. Liberibacter-psyllid combinations.

### Two *Wolbachia* strains reside in four psyllid species

The analysis identified two SVs corresponding to distinct lineages of *Wolbachia* (Alphaproteobacteria: Rickettsiales), which were previously detected in *D. citri* and *Diaphorina lycii* [30]. SV7, which was derived from 11.1% of denoised *C. biwa* reads, 9.9% of denoised *Cy. hexastigma* reads, and 31.1% of denoised *E. kuwayamai* reads (Supplementary Table 2), was 100% identical to the sequence of *Wolbachia*_iv previously identified in *D. citri* (TAAA01000013) collected in Japan. The sequence is also identical to those of *Wolbachia* detected in *D. citri* from the U.S.A. and the whitefly *Bemisia tabaci* (Hemiptera: Sternorrhyncha: Aleyrodoidea: Aleyrodidae) from various locations in the Asia-Pacific region [30]. SV43, derived from 1.1% of denoised *P. morimotoi* reads (Supplementary Table 2), was 100% identical to the sequence of *Wolbachia*_i previously identified in *D. citri* and *D. lycii* (TAAA01000005) [30]. The sequence is also identical to those of *Wolbachia* reported from the aphid *Cinara cedri* collected in Israel [102], and various insects in China, including the planthopper *Nilaparvata lugens* (Hemiptera: Auchenorrhyncha: Delphacidae), and aphids *Phloeomyzus passerinii* (Phloeomyzidae) and *Cervaphis quercus* (Aphididae) [103].

**Fig. 7:**
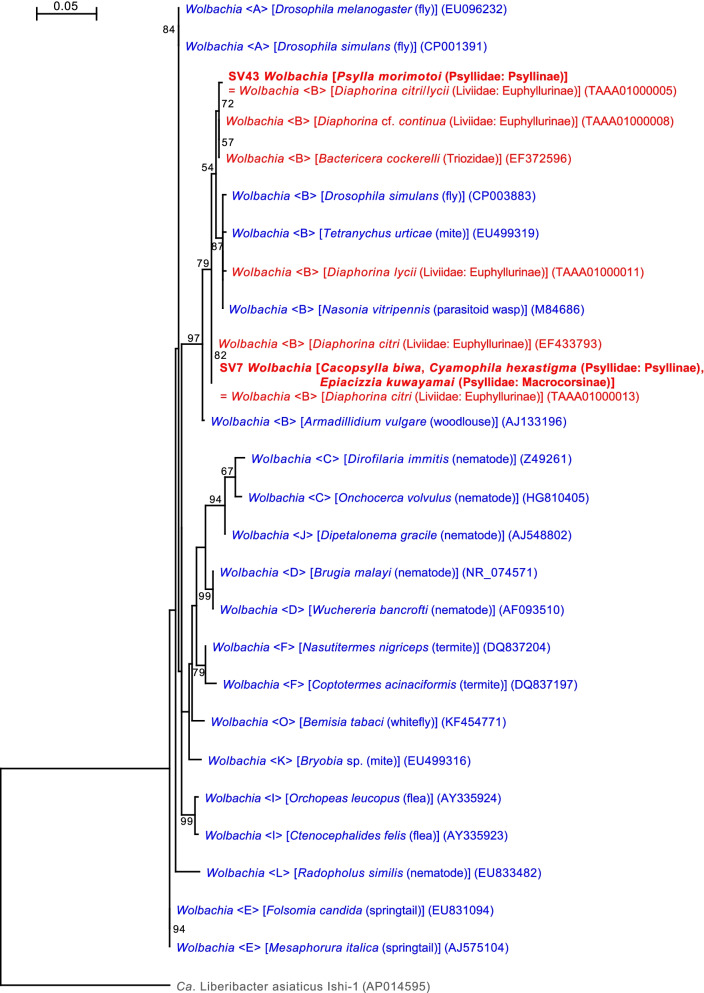
Maximum likelihood phylogram of *Wolbachia*. A total of 402 unambiguously aligned nucleotide sites of 16S rRNA genes were subjected to the analysis. On each branch, bootstrap support values of > 50% are shown. Host organisms are shown in brackets. Symbionts of animals other than psyllids are shown in blue. Symbionts of psyllids are shown in red. The sequence from this study is shown in bold. DDBJ/EMBL/GenBank accession numbers for sequences are provided in parentheses. Supergroups of *Wolbachia* are shown in angle brackets. The scale bar represents nucleotide substitutions per position. *Liberibacter* was used as an outgroup

*Wolbachia* are rickettsial bacteria distributed in a wide variety of arthropods and nematodes [104–106], whose strains are currently classified into supergroups A–Q [107]. Whereas supergroups A and B are monophyletic and are the most common supergroups infecting arthropods, supergroups C and D infect nematodes. Supergroups E–Q are found in various hosts, including nematodes, springtails, termites, fleas, aphids, and mites [106]. The molecular phylogenetic analysis placed SV7 and SV43 detected in the present study in the robustly supported clade of *Wolbachia* supergroup B (bootstrap: 97%) (Fig. 7). The majority of *Wolbachia* strains manipulate the reproduction of arthropod hosts through cytoplasmic incompatibility, feminization, male killing, and parthenogenesis to increase the prevalence of infected females in host populations [104–106]. Due to this ability to boost dissemination, *Wolbachia* are recognized to be promising agents to control insect pests by affecting their traits or microbiomes, including pathogens therein [108, 109]. Because of the high infection rates of *Wolbachia* in pest psyllids worldwide [18, 62, 66, 110–115], and the suggested interactions between *Wolbachia* and other symbionts [59–62, 116], the application of *Wolbachia* to control pest psyllids and/or plant pathogens is anticipated [59, 62, 111, 113, 115]. The present study suggests rampant horizontal transmissions of *Wolbachia* among various insect lineages, including pest psyllids, implying the feasibility of artificial infection and/or removal of *Wolbachia* in psyllids. Such techniques would facilitate the exploitation of *Wolbachia* as a tool to control pest psyllids and/or the plant pathogens they transmit.

### *Cacopsylla peninsularis* has *Rickettsia*

**Fig. 8:**
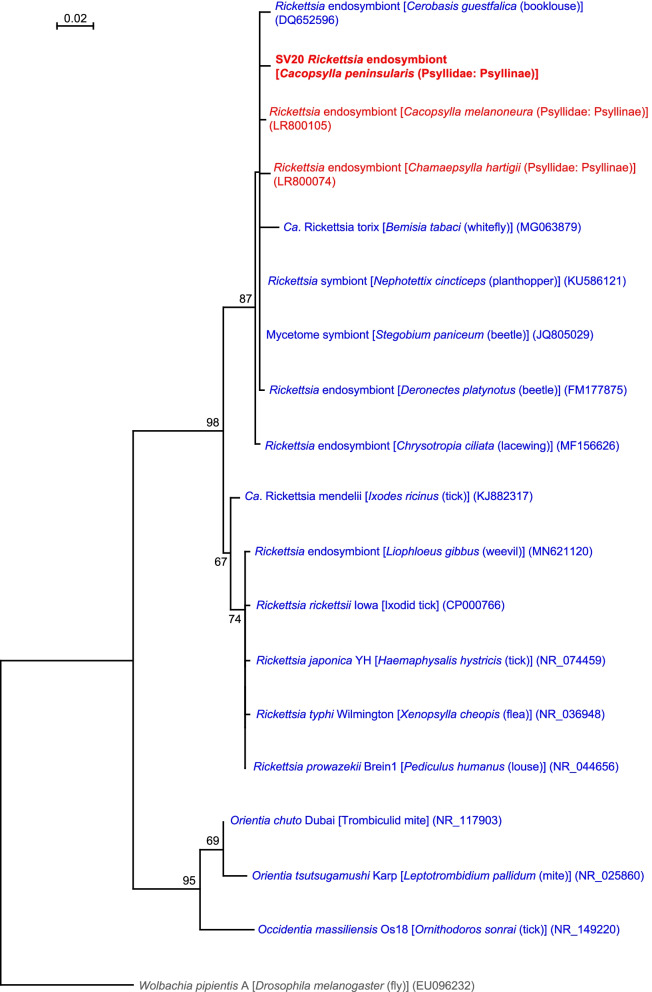
Maximum likelihood phylogram of *Rickettsia*. A total of 402 unambiguously aligned nucleotide sites of 16S rRNA genes were subjected to the analysis. On each branch, bootstrap support values of > 50% are shown. Host organisms are shown in brackets. Symbionts of animals other than psyllids are shown in blue. Symbionts of psyllids are shown in red. The sequence from this study is shown in bold. DDBJ/EMBL/GenBank accession numbers for sequences are provided in parentheses. The scale bar represents nucleotide substitutions per position. *Wolbachia* was used as an outgroup

The analysis detected *Rickettsia* sp. (Alphaproteobacteria: Rickettsiales) in *C. peninsularis*. SV20, which was derived from 31.4% of denoised *C. peninsularis* reads, was 99.5% identical to the sequence of ‘*Rickettsia* endosymbiont’ found in various arthropods including the planthopper *Nephotettix cincticeps* (Delphacidae) (KU586121). The sequence was 99.3 and 99.0% identical to ‘*Rickettsia* endosymbionts’ of the psyllids, *Cacopsylla melanoneura* (LR800105) and *Chamaepsylla hartigii* (LR800074) (Psyllidae: Psyllinae), respectively. Similar sequences were also detected from the drugstore beetle *Stegobium paniceum* (Coleoptera: Ptinidae) (JQ805029), the booklouse *Cerobasis guestfalica* (Psocoptera: Trogiidae) (DQ652596), the lacewing *Chrysotropia ciliata* (Neuroptera: Chrysopidae) (MF156626), and the whitefly *B. tabaci* (MG063879). These sequences formed a well-supported clade (bootstrap: 87%) in the ML tree (Fig. 8).

*Rickettsia* is closely related to *Wolbachia*, both of which belong to the order Rickettsiales. Similar to *Wolbachia*, some *Rickettsia* lineages cause reproductive disorders in host insects, including male killing and parthenogenesis [117, 118]. It would be worthwhile to assess whether *Rickettsia* endosymbionts manipulate the reproduction of psyllids, which will be potentially useful to exploit *Rickettsia* as a tool to control pest psyllids and/or plant pathogens.

## Conclusions

The present study found *Ca*. Fukatsuia symbiotica and *Serratia symbiotica*, which were recognized as aphid secondary symbionts, formally for the first time in Psylloidea. The analysis also found the potential plant pathogen, *Ca*. Liberibacter europaeus (Rhizobiales: Rhizobiaceae), for the first time in a pest psyllid feeding on the mulberry. Furthermore, *Wolbachia* and *Rickettsia*, plausible host reproduction manipulators, were detected among analyzed psyllids. The study also identified *Arsenophonus*, *Sodalis*, endosymbionts2, endosymbionts3, *Pseudomonas*, and *Diplorickettsia*, a plausible human pathogen. These findings suggest considerable interspecific transfer of arthropod symbionts, providing deeper insights into the evolution of interactions among insects, bacteria, and plants. This may be exploited to facilitate the control of pest psyllids with the aid of future studies to determine the localization and genomic structure of the identified bacteria.

## Methods

### Insects and DNA extraction

Adults of 12 psyllid species belonging to the family Psyllidae were collected from several trees of each host plant species in Japan (Table 1). Although most species were selected from the subfamily Psyllinae, *Epiacizzia kuwayamai* belongs to the subfamily Macrocorsinae [63]. We included this species because it originally belonged to Psyllinae with the name *Psylla kuwayamai* and its generic transfer from *Psylla* to *Epiacizzia* appeared to be inconsistent with the morphological features [119]. Also, whereas Burckhardt et al. recently proposed to transfer *Psylla morimotoi* (Psyllinae) to the genus *Spanioneura* (Psyllinae) based on its ecological features [2], the present study refers to this species as is because its morphological features are more consistent with the definition of *Psylla* (sensu stricto) [120, 121].

After surface sterilization with ethanol, DNA was extracted from the whole bodies of pooled individuals of five adult males and five adult females of each psyllid species using the DNeasy Blood & Tissue Kit (Qiagen, Hilden, Germany). The quality of the extracted DNA was assessed using a NanoDrop 2000c spectrophotometer (Thermo Fisher Scientific, Waltham, Massachusetts, U.S.A.) and the quantity was assessed using a Qubit 2.0 Fluorometer with the Qubit dsDNA HS Assay Kit (Thermo Fisher Scientific).

### Construction and sequencing of amplicon libraries

Bacterial populations in psyllids were analyzed using the MiSeq system (Illumina, San Diego, California, U.S.A.), as described previously [30]. Briefly, amplicon PCR was performed using the genomic DNA extracted from psyllids, the KAPA HiFi HotStart ReadyMix (KAPA Biosystems, Wilmington, Massachusetts, U.S.A.), and the primer set 16S_341Fmod (5′-TCGTCGGCAGCGTCAGATGTGTATAAGAGACAGYYTAMGGRNGGCWGCAG-3′) and 16S_805R (5′-GTCTCGTGGGCTCGGAGATGTGTATAAGAGACAGGACTACHVGGGTATCTAATCC-3′) targeting the V3 and V4 regions of the 16S rRNA gene. Whereas both primers were based on the instructions by Illumina [122], 16S_341F was modified (underlined), where original CC, C, and G were replaced with mixed bases, YY (C or T), M (A or C), and R (A or G). Our preliminary analyses demonstrated that this modification improves sensitivity to detect symbionts with AT-rich genomes including *Carsonella*, without reducing sensitivity for those with GC-rich genomes. Dual indices and Illumina sequencing adapters were attached to the amplicons with index PCR using the Nextera XT Index Kit v2 (Illumina). The libraries were combined with the PhiX Control v3 (Illumina), and 250 bp of both ends were sequenced on the MiSeq platform (Illumina) with the MiSeq Reagent Kit v2 (500 cycles; Illumina).

### Computational analysis of bacterial populations

After the amplicon sequence reads were demultiplexed, the output sequences were imported into the QIIME2 platform (version 2020.2) [123] and processed as described previously [30]. In brief, after primer sequences were removed using the cutadapt plugin [124], paired-end sequences were trimmed, denoised, joined, and dereplicated using the dada2 plugin [70]. During this step, chimeric sequences were detected and removed. The q2-feature-classifier plugin [125] was trained using the V3 and V4 regions of the 16S rRNA gene sequences retrieved from the SILVA database ver. 132 (SILVA_132_QIIME_release/taxonomy/16S_only/99/taxonomy_7_levels.txt) that were clustered at 99% sequence similarity [72]. Subsequently, the denoised and dereplicated amplicon reads were classified and taxonomic information was assigned using the trained q2-feature-classifier. Obtained sequence variants (SVs) were manually checked by performing BLASTN searches against the National Center for Biotechnology Information non-redundant database [126].

### Phylogenetic analysis of detected bacteria

Phylogenetic analysis of SVs was performed as described previously [30]. Briefly, after the SVs were aligned to related sequences using SINA (v1.2.11) [127], phylogenetic trees were inferred by the maximum likelihood (ML) method using RAxML (version 8.2.12) [128]. The GTR + Γ model was used with no partitioning of the data matrix, with 1000 bootstrap iterations (options -f a -m GTRGAMMA -# 1000).

## Supplementary Information

**Additional file 1.**

**Additional file 2.**

## Data Availability

The nucleotide sequence data are available in the DDBJ/EMBL/GenBank databases under the accession numbers DRR300288–DRR300299 (MiSeq output) and TAAB01000001–TAAB01000045 (dereplicated sequence variants).

## Abbreviations

*Ca*: *Candidatus*
SV: Sequence variant
ML: Maximum likelihood
*E*.: *Epiacizzia*
*C*.: *Cacopsylla*
*P*.: *Psylla*
PCR: Polymerase chain reaction
*A*: *Anomoneura*
*Cy*.: *Cyamophila*
*S*.: *Serratia*
*Pse*.: *Pseudomonas*
*M*.: *Macrosteles*
*D*.: *Diaphorina*
*L*: *Liberibacter*
*C*Leu: *Candidatus* Liberibacter europaeus
*Ar*.: *Arytainilla*
*B*.: *Bemisia*
